# Influence of *APOA5* Locus on the Treatment Efficacy of Three Statins: Evidence From a Randomized Pilot Study in Chinese Subjects

**DOI:** 10.3389/fphar.2018.00352

**Published:** 2018-04-11

**Authors:** Sha Hua, Chuanxiang Ma, Jun Zhang, Jing Li, Weiwei Wu, Ning Xu, Guanghua Luo, Jianrong Zhao

**Affiliations:** ^1^Department of Cardiology, Ruijin Hospital Luwan Branch, School of Medicine, Shanghai Jiao Tong University, Shanghai, China; ^2^Department of Pathology, Affiliated Hospital of Weifang Medical University, Weifang, China; ^3^Comprehensive Laboratory, Third Affiliated Hospital of Soochow University, Changzhou, China; ^4^Section of Clinical Chemistry and Pharmacology, Institute of Laboratory Medicine, Lund University, Lund, Sweden

**Keywords:** *APOA5* genotype, statins, triglycerides, LDL cholesterol, dyslipidemia

## Abstract

Pharmacogenetics or pharmacogenomics approaches are important for addressing the individual variabilities of drug efficacy especially in the era of precision medicine. One particular interesting gene to investigate is *APOA5*, which has been repeatedly linked with the inter-individual variations of serum triglycerides. Here, we explored *APOA5*-statin interactions in 195 Chinese subjects randomized to rosuvastatin (5–10 mg/day), atorvastatin (10–20 mg/day), or simvastatin (40 mg/day) for 12 weeks by performing a targeted genotyping analysis of the *APOA5* promoter SNP rs662799 (-1131T > C). There were no significant differences between the treatment arms for any of the statin-induced changes in clinical biomarkers. Reductions in LDL cholesterol were influenced by the *APOA5* genotype in all three treatment groups. By contrast, changes in HDL cholesterol, and triglycerides were only affected by the *APOA5* genotype in the atorvastatin and simvastatin groups and not in the rosuvastatin group. Our results suggest that future studies may need to consider stratifying subjects not only by genetic background but also by prescribed statin type.

## Introduction

Statins are the most prescribed class of drugs worldwide for prevention of various cardiovascular diseases. However, about one third of patients do not respond well to this therapy with respect to the lipid-lowering effect, suggesting that pharmacogenomics (Postmus et al., 2014) or other environmental factors such as diet (Jenkins et al., 2005) or the gut microbiome (Kaddurah-Daouk et al., 2011) may play substantial roles. To date, genome-wide association studies have identified at least 39 genes that are associated with statin treatment efficacy (Gryn and Hegele, 2014). Most of these genes are involved in either the direct pharmacokinetic handling of statins or in lipid metabolism pathways especially those involving cholesterol, the main target of statin therapy (Mangravite et al., 2006). However, accumulating evidence indicates that statins can also lower levels of triglycerides, potentially through altering degradation of apolipoprotein B (ApoB) and related very low-density lipoprotein (VLDL) balance, although the precise mechanism remains unclear (Ginsberg et al., 1987; Arad et al., 1992; Ginsberg, 1998).

One gene of particular interest within this context is *APOA5* (Guardiola and Ribalta, 2017), which was identified in 2001 (Pennacchio et al., 2001; van der Vliet et al., 2001) and has been associated with high inter-individual variations of serum triglycerides in all reported populations (Baum et al., 2003; Lai et al., 2004; Hubacek et al., 2008; Ouatou et al., 2014; Son et al., 2015). Accumulating evidence also suggests that polymorphisms in this gene confers risk for cardiovascular disease (Lai et al., 2004; Han et al., 2017) and myocardial infarction (Do et al., 2015). Two of the most characterized *APOA5* SNPs are rs662799 (-1131T > C) and rs3135506 (56C > G) (Hubacek et al., 2004; Lim et al., 2014; Chen et al., 2018; Kim et al., 2018). Of note, rs662799 is more common in the Asian population (26–40%) than in Caucasians (∼8%) (Baum et al., 2003; Kim et al., 2018). According to one estimation, this SNP alone can contribute to 6.2% of the genetic component of hypertriglyceridemia (Hegele, 2009).

Although it has previously been suggested that there is a link between *APOA5* and statin treatment (Brautbar et al., 2011; O’Brien et al., 2015), neither the type nor the dose of statins was taken into consideration in these earlier studies; it is important to note that statins differ in terms of their pharmacodynamic and pharmacogenetic properties (Kivisto et al., 2004; Schachter, 2005) and potency (Palmer et al., 2013; Arshad, 2014; Karlson et al., 2016). Another retrospective study did not observe an effect of statin type when investigating the interaction between the rs662799 variants and statins (Hubacek et al., 2009); however, this study did not include rosuvastatin, which is often considered to be a better treatment choice (Scott et al., 2004; McKenney, 2005; Schachter, 2005).

Here, we performed a pilot study to explore *APOA5*-statin interactions in 195 Chinese subjects randomized to rosuvastatin, atorvastatin, or simvastatin therapy for 12 weeks. To address whether the reduction in cholesterol and apolipoprotein levels of three types of statins differ between subjects with the same *APOA5* genetic background, we genotyped the *APOA5* rs662799 SNP and measured the fasting plasma concentrations of triglycerides, cholesterols, FFAs, and four apolipoproteins both before and after statin treatments.

## Materials and Methods

### Study Subjects and Study Design

Between 2015 and 2016, we screened 240 patients with treatment-naive dyslipidemia at Shanghai Ruijin Hospital Luwan Branch (affiliated to Shanghai Jiao Tong University) and recruited 195 to this study (Supplementary Figure S1). The inclusion criteria were: (i) preregistered with our medial examination center for annual routine heath screening; (ii) aged 18 years or older; (iii) newly diagnosed with dyslipidemia and/or increased risk of atherosclerotic cardiovascular diseases and recommended to receive statins according to the 2013 American College of Cardiology and the American Heart Association Blood Cholesterol Guidelines (Stone et al., 2014); (iv) capable of understanding the scope and potential consequences of this study. The exclusion criteria were: (i) major systematic diseases such as malignancy; (ii) heart failure experience; (iii) dramatic weight loss and medication (especially antibiotics) 2 months before recruitment except antihypertensive therapy; (iv) acute illnesses. All patients newly diagnosed with dyslipidemia were encouraged to adopt lifestyle changes first for 2–4 months before moderate statin treatment; thus, only those who failed to achieve the therapeutic goal (mostly due to poor adherence to the dietary and/or exercise recommendations) were considered as eligible for this study.

The 195 subjects were then randomly divided into three treatment arms to receive rosuvastatin (5–10 mg/day), atorvastatin (10–20 mg/day), or simvastatin (40 mg/day) for 12 weeks (*n* = 65 for each treatment arm). To achieve comparable clinical efficacies in response to the three statins, the different statin doses were selected based on both clinical practice and evidence suggesting that each rosuvastatin dose is equivalent to 3–3.5 times of atorvastatin and 7–8 times of simvastatin (at least in terms of cholesterol reduction) (Hubacek et al., 2009). Follow-up evaluations were performed every 2 weeks and no side effects or poor adherence was reported.

Written informed consent was obtained from all the study participants. This study conforms to the ethical guidelines of the 1975 Declaration of Helsinki and was approved by Ethics Committee of Shanghai Ruijin Hospital Luwan Branch. Complete clinical trial registration is deposited at chictr.org.cn (ChiCTR-RRC-16010131).

### Laboratory Analyses

Fasting plasma concentrations of triglycerides, total cholesterol, HDL cholesterol (HDLc), LDL cholesterol (LDLc), FFAs, three different apolipoproteins (ApoA1, ApoB-100, and ApoE), and Lp(a) were measured by enzymatic methods using a Beckman Coulter Chemistry Analyzer AU5800 Series (United States) at both baseline and 12 weeks after treatments.

After the randomization and initiation of treatments, DNA was isolated using the TIANamp Blood DNA kit (purchased from Tiangen, Beijing, China) and individual *APOA5* variants (-1131T > C – rs662799) were genotyped using a base-quenched probe method combined with polymerase chain reaction (PCR) as described before (Luo et al., 2009). In brief, a 19-nt probe (5′-GGCAAATCTCACTTTCGCT-3′) containing the targeted SNP site was first conjugated with 6-carboxyfluorescein and then hybridized to its complementary target sequence from PCR amplification. An analytical melting program that involves heating the amplicon/probe heteroduplex will produce different fluorescence curves depending on the genotypes of rs662799. Both the probe and primers (forward: 5′-AGGAGTGTGGTAGAAAGACCTGTTG-3′; reverse: 5′-AACTACCCAGAGTCACTGTGTCCC-3′) used were synthesized by Sangon (Shanghai, China).

### Statistical Analysis

Statistical differences between groups were estimated by Wilcox rank-sum test (between two groups), Kruskal–Wallis test (among three groups) for continuous variables or by Chi-square test for categorical variables. T/C and C/C subjects were pooled as C allele carriers for all statistical analyses to increase the power. Both the percentage and absolute changes of LDLc, HDLc and triglycerides in response to each statin were further adjusted for age, sex, and BMI by using linear regression models. In cases of models based on absolute changes, the baseline values were also entered as covariates. However, statin doses were not considered since similar doses of statin were prescribed for all subjects within the same treatment arm. A backward variable selection procedure was performed using R function ‘step’ based on the Akaike information criterion (AIC). *APOA5*-statin interaction was further explored by adding an interaction term into the models. Associations between apolipoproteins and concentrations of LDLc and HDLc were measured by Spearman’s rank correlation analysis. Hardy–Weinberg Equilibrium was accessed by exact test based on R package “HardyWeinberg” (Graffelman, 2015). Raw P values were adjusted by the Benjamini–Hochberg method (Benjamini and Hochberg, 1995) with a false discovery rate of 5%. A power of 99.98% was obtained using pwr package (Champely, 2015) for this study based on 65 patients (sample size for each treatment arm) with paired design, 5% significance, and an estimated effect size of 0.7 (i.e., effective in 70% of the patients) for statins to reduce LDL cholesterol (Cholesterol Treatment Trialists (CTT) Collaborators, 2012; Ridker et al., 2016). All statistical tests and data visualizations as well as the stratified randomization process by considering BMI as covariate were performed under the R environment (R Development Core Team, 2015).

## Results

### Baseline Characteristics

The minor C allele frequency of *APOA5* rs662799 SNP in our cohort was 30%, consistent with other reports based on larger Chinese cohorts (Baum et al., 2003; Jiang et al., 2010); the genotype frequency of *APOA5* was in agreement with Hardy–Weinberg Equilibrium (*n* = 13, 91 and 91 for C/C, T/C, and T/T allele carriers, respectively; *P* = 0.171). With the exception of ApoE, there were no significant baseline differences between the treatment arms, including the frequencies of the three *APOA5* genotypes (*P* = 0.597) (**Table 1**). These data suggest that the treatment groups are in general homogeneous and this study design is suitable for addressing the relationship between *APOA5* variations and the clinical responses of three statins.

**Table 1:** Baseline characteristics summarized by statin treatment and *APOA5* genotypes, respectively.

Characteristics	Statin treatment^a^				*APOA5* genotype^a^			
	Atorvastatin	Rosuvastatin	Simvastatin	*P*^b^	C/C	T/C	T/T	*P*^e^
*n*	4/32/29^d^	5/25/35^d^	4/34/27^d^	0.597^c^	13	91	91	-
Male (%)	53.8	44.6	49.2	0.575^c^	69.2	41.8	53.8	0.288^c^
Age (years)	74.9 ± 10.5	75.0 ± 11.8	69.8 ± 17.4	0.344	72.2 ± 13.9	72.9 ± 12.7	73.7 ± 14.8	0.420
BMI (kg/m^2^)	23.5 ± 3.2	23.5 ± 3.3	23.4 ± 3.2	0.938	24.1 ± 2.7	23.4 ± 3.7	23.3 ± 2.8	0.662
Tc (mg/dl)	193.1 ± 49.5	180.2 ± 40.6	186.7 ± 49.0	0.306	194.1 ± 28.2	191.6 ± 47.3	180.7 ± 47.6	0.093
Tg (mg/dl)	180.1 ± 110.5	162.6 ± 102.6	181.9 ± 138.0	0.482	266.9 ± 151.2	183.3 ± 117.8	153.4 ± 105.8	0.004
HDLc (mg/dl)	42.1 ± 9.9	44.1 ± 10.4	44.6 ± 11.5	0.458	41.1 ± 11.3	43.4 ± 10.7	44.1 ± 10.6	0.513
LDLc (mg/dl)	132.2 ± 42.9	125.8 ± 34.8	125.7 ± 41.1	0.780	145.4 ± 35.0	133.1 ± 41.3	120.1 ± 37.3	0.015
ApoA1 (mg/dl)	114.4 ± 20.6	119.1 ± 23.3	116.9 ± 20.1	0.515	113.4 ± 8.8	116.7 ± 23.2	117.3 ± 20.7	0.708
ApoB-100 (mg/dl)	86.3 ± 31.2	86.5 ± 25.0	94.1 ± 29.2	0.181	99.5 ± 22.2	91.0 ± 29.3	85.4 ± 28.5	0.145
ApoE (mg/dl)	4.4 ± 1.5	3.8 ± 1.0	4.5 ± 1.4	0.019	4.7 ± 1.7	4.3 ± 1.3	4.2 ± 1.3	0.382
FFA (mmol/l)	0.5 ± 0.2	0.5 ± 0.3	0.5 ± 0.2	0.780	0.5 ± 0.1	0.5 ± 0.2	0.4 ± 0.3	0.052
Lp(a) (mg/dl)	17.5 ± 17.0	18.0 ± 15.4	19.8 ± 17.9	0.916	25.3 ± 25.2	17.5 ± 12.6	18.4 ± 18.8	0.575
Type 2 diabetes (%)	43.1	40.0	32.3	0.430^c^	53.8	38.5	36.3	0.658^c^

^a^*Continuous variables are expressed as mean ± standard deviations (sd); ^*b*^*P*-values were estimated by Krukskal–Wallis test for continuous variables; ^*c*^*P*-values were estimated by Chi-square test for categorical variables; ^*d*^Sample sizes for individuals genotyped as C/C, T/C and T/T, respectively; ^*e*^*P*-values were estimated by Wilcox rank-sum test for continuous variables (C/C and T/C were pooled as one group in this case)*.

When stratifying the subjects by genotype, the C allele carriers (including both C/C and T/C) had significantly higher plasma triglycerides than T/T carriers at baseline (**Table 1**), in agreement with previous studies (Baum et al., 2003; Lai et al., 2004; Jiang et al., 2010). We also noted that subjects with the C allele carriers had higher LDLc than T/T carriers at baseline (**Table 1**).

### Rosuvastatin-Induced Changes in HDLc and Triglycerides Are Not Associated With *APOA5* Genotype

**FIGURE 1:**
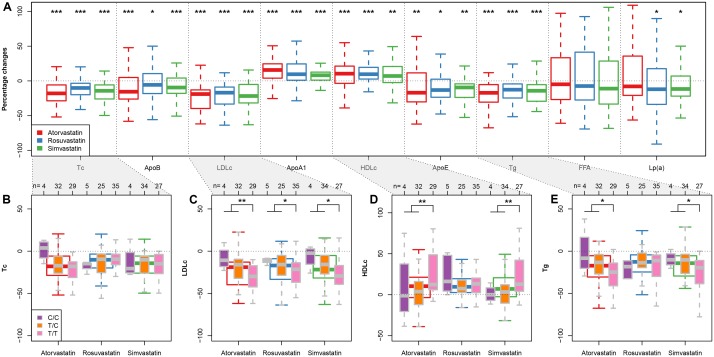
*APOA5*-statin interactions. **(A)** Box plots (with median) showing percentage changes in the indicated biomarkers after treatment with rosuvastatin (5–10 mg/day), atorvastatin (10–20 mg/day) or simvastatin (40 mg/day). ^∗^*P* < 0.05; ^∗∗^*P* < 0.01; ^∗∗∗^*P* < 0.001 versus before treatment. (Wilcoxon signed-rank test) **(B–E)** Box plots (with median) showing percentage changes in total cholesterol (Tc) **(B)**, LDLc **(C)**, HDLc **(D)**, and triglycerides (Tg) **(E)** in response to each statin in subjects divided by *APOA5* rs662799 genotypes. Sample sizes for each subgroup are given on top of **(B–E)**. ^∗^*P* < 0.05; ^∗∗^*P* < 0.01 (Wilcoxon rank-sum test; C/C and T/T subjects were pooled together for statistical testing).

We next compared the clinical efficacies of the statins (in terms of changes of cholesterol, triglyceride, and apolipoprotein). As expected, all three statins promoted significant reductions in total cholesterol, ApoB, LDLc, ApoE, and triglycerides and significant increases in ApoA1 and HDLc (**Figure 1A**). However, there were no significant differences between the treatment arms for any of the statin-induced changes in clinical biomarkers after adjusting for multiple testing (false discovery rate 5%), confirming that the response to 5–10 mg of rosuvastatin is similar to that of 10–20 mg atorvastatin and 40 mg of simvastatin as suggested previously (Hubacek et al., 2009). In agreement, results from a meta-analysis (Karlson et al., 2016), comparative pharmacology (McTaggart, 2003) and the MERCURY II clinical trial (Ballantyne et al., 2006) have all shown that rosuvastatin is more potent than the other statins and thus lower doses can be used to achieve equivalent responses.

To determine how *APOA5* variations were associated with the clinical responses of the three statins, we investigated how changes in the biomarker concentrations in response to each statin varied among the three *APOA5* genotypes (**Figures 1B–E** and Supplementary Table S1). No significant differences were observed among three *APOA5* variants in terms of the percentage changes of total cholesterol (**Figure 1B**), apolipoproteins, FFA, and Lp(a) (data not shown) in response to any of the three statins. However, compared with the C allele carriers, patients homozygous for the major T allele exhibited: (1) lower baseline LDLc levels independent of statin type (**Table 1**); (2) significantly larger statin-induced LDLc reductions, independent of statin type (**Figure 1C**); and (3) more pronounced statin-induced changes in HDLc and triglycerides upon atorvastatin or simvastatin treatment (**Figures 1D,E**). By contrast, rosuvastatin-induced changes in HDLc and triglycerides showed little variation among the three *APOA5* variants (**Figures 1D,E**). This finding suggesting that statin–*APOA5* interactions may also depend on the statin type was further supported by linear regression analyses by adding the treatment and genotype interaction term (**Table 2**); the results were still valid after adjusting for age, sex, and BMI (**Table 2**). We obtained similar results when using the absolute changes of each biomarker additionally adjusted for the baseline biomarker values (Supplementary Table S2).

**Table 2:** Percentage changes of LDLc, HDLc, and triglycerides in response to each statin treatment adjusted for sex, age, and BMI using linear regression models.

Initial models	Treatment	Final models
%LDLc ∼ sex+age+BMI+genotype	Atorvastatin	%LDLc ∼ genotype
		*P*_model_ = 0.003
		***P*_genotype_ = 0.003**
	Rosuvastatin	%LDLc ∼ sex+BMI+genotype
		*P*_model_ = 0.015
		***P*_genotype_ = 0.046**
	Simvastatin	%LDLc ∼ sex+age+genotype
		*P*_model_ = 0.002
		***P*_genotype_ = 0.02**
%LDLc ∼ sex+age+BMI+genotype+treatment+interaction	Interaction	%LDLc ∼ age+genotype
%HDLc ∼ sex+age+BMI+genotype	Atorvastatin	%HDLc ∼ age+genotype
		*P*_model_ = 0.0004
		***P*_genotype_ = 0.002**
	Rosuvastatin	Not significant
	Simvastatin	%HDLc ∼ genotype
		*P*_model_ = 0.002
		***P*_genotype_ = 0.002**
%HDLc ∼ sex+age+BMI+genotype+treatment+interaction	Interaction	%HDLc ∼ age+genotype+treatment+interaction
%Tg ∼ sex+age+BMI+genotype	Atorvastatin	%Tg ∼ genotype
		*P*_model_ = 0.01
		***P*_genotype_ = 0.01**
	Rosuvastatin	%Tg ∼ BMI
		*P*_model_ = 0.01
	Simvastatin	%Tg ∼ genotype
		*P*_model_ = 0.006
		***P*_genotype_ = 0.006**
%Tg ∼ sex+age+BMI+genotype+treatment+interaction	Interaction	%Tg ∼ BMI+genotype+treatment+interaction

### Statin–*APOA5* Interactions Altered the Correlations Between Apolipoproteins and LDLc/HDLc

**FIGURE 2:**
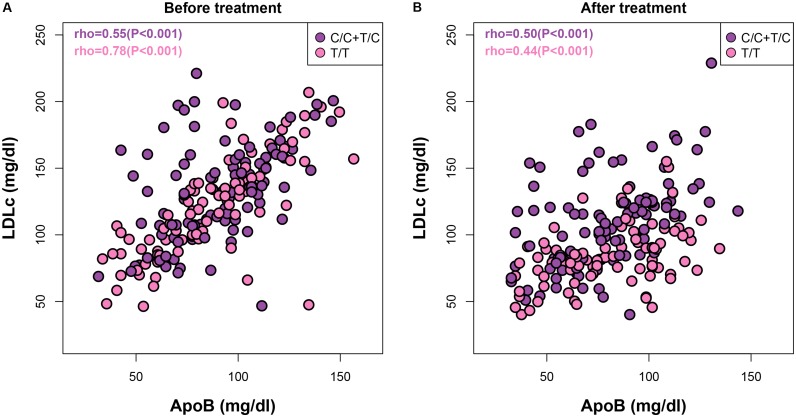
**|** Both *APOA5* and statin alter the ApoB–LDLc correlations. Correlations between ApoB and LDLc before **(A)** and after **(B)** statin treatment in subjects with *APOA5* rs662799 C or T/T allele.

Although most therapies to reduce cardiovascular disease risk currently focus on reduction of LDLc and triglycerides, atherogenic proteins such as ApoB have also been suggested to have great predictive value (Ballantyne et al., 2008). Accordingly, the American Diabetes Association and the American College of Cardiology Foundation recommend that therapy for patients with high cardiovascular disease risk should aim to lower ApoB concentrations to below 90 mg/dl in addition to reducing LDLc levels (Brunzell et al., 2008). To address whether the well-known associations between ApoB and LDLc both before and after statin treatments (Ballantyne et al., 2008) differ among patients with different *APOA5* genotypes, we additionally analyzed ApoB–LDLc correlations within each *APOA5* SNP subgroup. For C allele carriers, the Spearman correlation coefficients between ApoB and LDLc were comparable before and after treatment [ρ = 0.55 (*P* < 0.001) and 0.50 (*P* < 0.001), respectively] (**Figure 2**). By contrast, in T/T homozygotes, the ApoB–LDLc correlation reduced dramatically after treatment [from 0.78 (*P* < 0.001) before treatment to 0.44 (*P* < 0.001)], indicating that the statin-induced reduction of ApoB in absolute values was much smaller than the statin-induced reduction of LDLc in these patients. Thus, further treatment to reduce the levels of ApoB even after achieving recommended LDLc reductions could be beneficial in T/T carriers. Similar observations were found between ApoA1 and HDLc (Supplementary Figure S2).

## Discussion

Our pilot study revealed that rosuvastatin achieved comparable improvements in all biomarkers examined despite the fact that it was prescribed at a much lower dose than atorvastatin and simvastatin. We further demonstrated that the reduction of LDLc was strongly affected by *APOA5* independent of the statin type prescribed. In contrast, the percentage changes of HDLc and triglycerides were less affected by *APOA5* variants in the rosuvastatin group than in the other two treatment arms.

Our findings about the strong *APOA5*-LDLc interactions are consistent with observations in a larger cohort (Lai et al., 2004) but not with an earlier study involving only Chinese men (Baum et al., 2003). It is not clear how *APOA5* variants affect LDLc as ApoA5 has only been detected on HDL and VLDL and not on LDL particles (Ballantyne et al., 2006). However, ApoA5 has been shown to directly interact with members of the LDL-receptor family (Nilsson et al., 2007). In addition, an earlier study has shown a significant association between the *APOA5* rs662799 SNP and increased risk of early-onset myocardial infarction even after adjusting for triglycerides (De Caterina et al., 2011), providing further evidence that this SNP may simultaneously affect other atherogenic lipids such as LDLc. It is also possible that this SNP is in complete linkage disequilibrium with other polymorphism(s) that can explain the observed LDLc levels.

In contrast to LDLc, we observed significant *APOA5*-HDLc and -triglyceride interactions in the atorvastatin and simvastatin groups but not in the rosuvastatin group. Possible explanations for these different treatment responses according to genotype include the following: (1) the hydrophilic rosuvastatin is largely excreted unchanged (Martin et al., 2003) whereas the other two lipophilic statins undergo substantial metabolism by the CYP450 pathways and thus are potentially more affected by gene polymorphisms (Kivisto et al., 2004; Schachter, 2005); (2) rosuvastatin differs from the other statins by its stronger binding to 3-hydroxy-3-methylglutaryl coenzyme A (HMG-CoA) reductase, lower systemic bioavailability, longer elimination half-life (McTaggart, 2003) and greater hepatoselectivity (Schachter, 2005); (3) an involvement of other apolipoproteins as *APOA5* variants have been associated with modulations of lipoprotein subclasses especially VLDL and HDL; however, no link between *APOA5* and ApoB has been reported (Talmud et al., 2004; Guardiola et al., 2015; Guardiola and Ribalta, 2017); and (4) an involvement of other SNPs, such as the *APOA5* variant rs2266788, that are in strong linkage disequilibrium with rs662799 (Caussy et al., 2014). To fully understand how *APOA5* affects statin treatments, in-depth characterizations of its functional role are still needed.

Our findings in this pilot study are limited by both the sample size and targeted genotyping of a single SNP. A further limitation is that we did not measure ApoA5 protein levels; however, previous studies have suggested that the *APOA5* SNP rs662799 studied here is not associated with *APOA5* mRNA expression levels or with circulating concentrations of this apolipoprotein (Talmud et al., 2006; Henneman et al., 2007; Kim et al., 2018). Some of the participants did not respond to statins, and we cannot exclude the possibility that poor adherence to their prescribed treatment played a role. However, good adherence to statin treatment was reported.

## Conclusion

In summary, our results show that low-dose rosuvastatin achieves improvements in clinical responses that are comparable to those observed with higher doses of atorvastatin and simvastatin but are less affected by *APOA5* genotype. These findings support the growing recognition that rosuvastatin is a potentially better treatment option for patients with dyslipidemia and/or at high risk of cardiovascular diseases when genetic information is not available. In addition, integrated efforts, such as the NIH Pharmacogenetics Research Network (Giacomini et al., 2007), should be encouraged in the era of precision medicine to accelerate pharmacogenetics or pharmacogenomics research. Future studies may also need to consider stratifying populations by genetic background and by prescribed statin type.

## Author Contributions

SH, GL, NX, and JrZ designed the study. SH performed the randomization process and clinical intervention. JL and WW enrolled participants and measured the lipids and apolipoproteins. JZ and GL performed the genotyping analysis. SH, CM, JL, and WW collected and analyzed the data. SH, CM, GL, and JrZ wrote the manuscript.

## Conflict of Interest Statement

The authors declare that the research was conducted in the absence of any commercial or financial relationships that could be construed as a potential conflict of interest.

## Abbreviations

ApoA5: apolipoprotein A5
BMI: body mass index
FFAs: free fatty acids
HDLc: high-density lipoprotein cholesterol
LDLc: low-density lipoprotein cholesterol
Lp(a): lipoprotein(a)
SNP: single nucleotide polymorphism
T2D: type 2 diabetes
Tc: total cholesterol
Tg: triglycerides.
