# Supplementary material for: Influence of APOA5 Locus on the Treatment Efficacy of Three Statins: Evidence From a Randomized Pilot Study in Chinese Subjects
Source: Front Pharmacol. 2018 Apr 11;9:352. doi: 10.3389/fphar.2018.00352 (PMC5904201; doi:10.3389/fphar.2018.00352)
Supplement: Supplementary file 1 [file Table_1.PDF]

Supplementary Table S1. Absolute values for all biomarkers examined both before and after statin treatments.

Biomarker (mg/dl)	Genotype	Atorvastatin Before	Atorvastatin After	Rosuvastatin Before	Rosuvastatin After	Simvastatin Before	Simvastatin After
Tc	C/C	202.1±26.4	204.1±30.1	184.8±26.7	157.1±39.0	197.7±36.0	175.0±72.2
	T/C	201.1±55.4	164.3±42.5	180.0±38.3	151.4±37.4	191.2±44.3	157.1±35.8
	T/T	183.1±43.9	143.8±39.2	179.8±44.6	160.3±35.2	179.4±56.3	147.6±49.6
LDLc	C/C	145.7±12.5	132.3±13.0	153.8±42.9	136.4±41.5	134.7±44.9	126.3±49.1
	T/C	136.7±49.8	108.1±40.1	126.8±32.5	100.9±26.1	134.5±38.9	109.4±29.1
	T/T	125.4±36.8	83.4±20.2	121.0±34.4	89.1±23.5	113.3±41.5	78.7±23.7
ApoB	C/C	92.8±16.4	78.5±29.4	109.6±27.1	81.8±15.1	93.8±21.2	86.5±17.7
	T/C	90.6±32.1	75.9±28.1	85.2±24.2	78.5±24.4	95.8±30.0	85.0±24.8
	T/T	80.7±31.5	70.4±28.3	84.2±24.3	83.1±23.7	92.1±29.8	83.1±24.6
ApoE	C/C	4.5±0.7	4.4±0.9	3.6±0.7	3.2±1.0	6.3±2.2	5.1±2.7
	T/C	4.4±1.6	3.9±1.7	3.8±0.7	3.7±1.3	4.6±1.2	4.3±0.9
	T/T	4.4±1.4	3.9±1.4	3.9±1.2	3.7±1.4	4.2±1.4	3.3±0.7
HDLc	C/C	41.3±18.7	39.9±9.0	39.2±8.8	47.7±9.3	43.3±6.9	43.4±6.8
	T/C	42.9±10.4	43.5±9.3	43.2±12.5	45.4±13.6	44.0±9.7	44.7±12.3
	T/T	41.4±8.2	50.7±11.9	45.5±8.9	50.5±12.2	45.4±14.1	52.9±14.2
ApoA1	C/C	114.3±8.4	113.5±20.5	111.4±4.9	126.8±8.8	115.0±14.1	129.8±14.9
	T/C	114.6±22.7	131.0±23.5	118.0±26.9	124.4±29.5	117.8±21.3	123.0±25.9
	T/T	114.1±19.9	135.0±21.8	120.9±22.1	136.8±23.3	116.0±19.8	129.7±23.3
Tg	C/C	254.9±173.5	218.8±98.8	232.6±116.1	163.1±107.6	321.7±192.9	299.1±190.6
	T/C	200.9±127.5	168.3±123.6	183.4±125.6	158.9±106.4	166.6±102.7	143.9±83.9
	T/T	146.9±65.4	98.7±36.2	137.8±73.8	101.3±38.1	180.5±160.9	98.2±40.8
FFA	C/C	0.5±0.1	0.5±0.3	0.5±0.1	0.4±0.2	0.4±0.1	0.3±0.0
	T/C	0.5±0.2	0.5±0.2	0.5±0.2	0.5±0.2	0.5±0.2	0.4±0.2
	T/T	0.4±0.2	0.4±0.2	0.5±0.4	0.4±0.2	0.4±0.2	0.4±0.2
Lp(a)	C/C	24.4±1.1	20.9±2.2	14.4±15.3	16.1±13.2	39.9±41.7	44.2±55.1
	T/C	17.8±12.6	17.6±13.3	16.8±13.1	15.2±13.8	17.6±12.7	15.7±11.3
	T/T	16.2±21.9	18.7±24.5	19.3±17.1	16.2±15.2	19.6±17.8	17.2±15.8
