# Supplementary material for: Influence of APOA5 Locus on the Treatment Efficacy of Three Statins: Evidence From a Randomized Pilot Study in Chinese Subjects
Source: Front Pharmacol. 2018 Apr 11;9:352. doi: 10.3389/fphar.2018.00352 (PMC5904201; doi:10.3389/fphar.2018.00352)
Supplement: Supplementary file 2 [file Table_2.PDF]

Supplementary Table S2. Linear regression models based on absolute values.

Initial models	Treatment	Final models
step(lm(ΔLDLc~baseline+Gender+Age+BMI+Genotype),direction="backward")	Atorvastatin	ΔLDLc~baselineLDLc+Genotype
		P_{model} = 1.441e-09
		Pgenotype= 0.0005
	Rosuvastatin	ΔLDLc~baselineLDLc+Gender+BMI+Genotype
		P_{model} = 9.827e-09
		$P_{genotype}$= 0.006
	Simvastatin	ΔLDLc~baselineLDLc+Gender+Genotype
		P_{model} = 2.209e-10
		$P_{genotype}$= 6.23e-05
step(lm(ΔHDLc ~baseline+Gender+Age+BMI+Genotype),direction="backward")	Atorvastatin	ΔHDLc~baselineHDLc+Age+Genotype
		P_{model} = 4.537e-06
		$P_{genotype}$= 0.0003
	Rosuvastatin	Not significant
	Simvastatin	ΔHDLc~baselineHDLc+Gender+Genotype
		P_{model} = 0.0003
		$P_{genotype}$= 0.01
step(lm(ΔTg ~baseline+Gender+Age+BMI+Genotype),direction="backward")	Atorvastatin	ΔTg~baselineTg+Genotype
		P_{model} = 5.464e-05
		$P_{genotype}$= 0.008
	Rosuvastatin	ΔTg~baselineTg+BMI
		P_{model}=5.027e-09
	Simvastatin	ΔTg~baselineTg+BMI+Genotype
		P_{model} = 1.561e-14
		$P_{genotype}$= 0.0002
