# Supplementary figures and images for: Influence of APOA5 Locus on the Treatment Efficacy of Three Statins: Evidence From a Randomized Pilot Study in Chinese Subjects
Source: Front Pharmacol. 2018 Apr 11;9:352. doi: 10.3389/fphar.2018.00352 (PMC5904201; doi:10.3389/fphar.2018.00352)

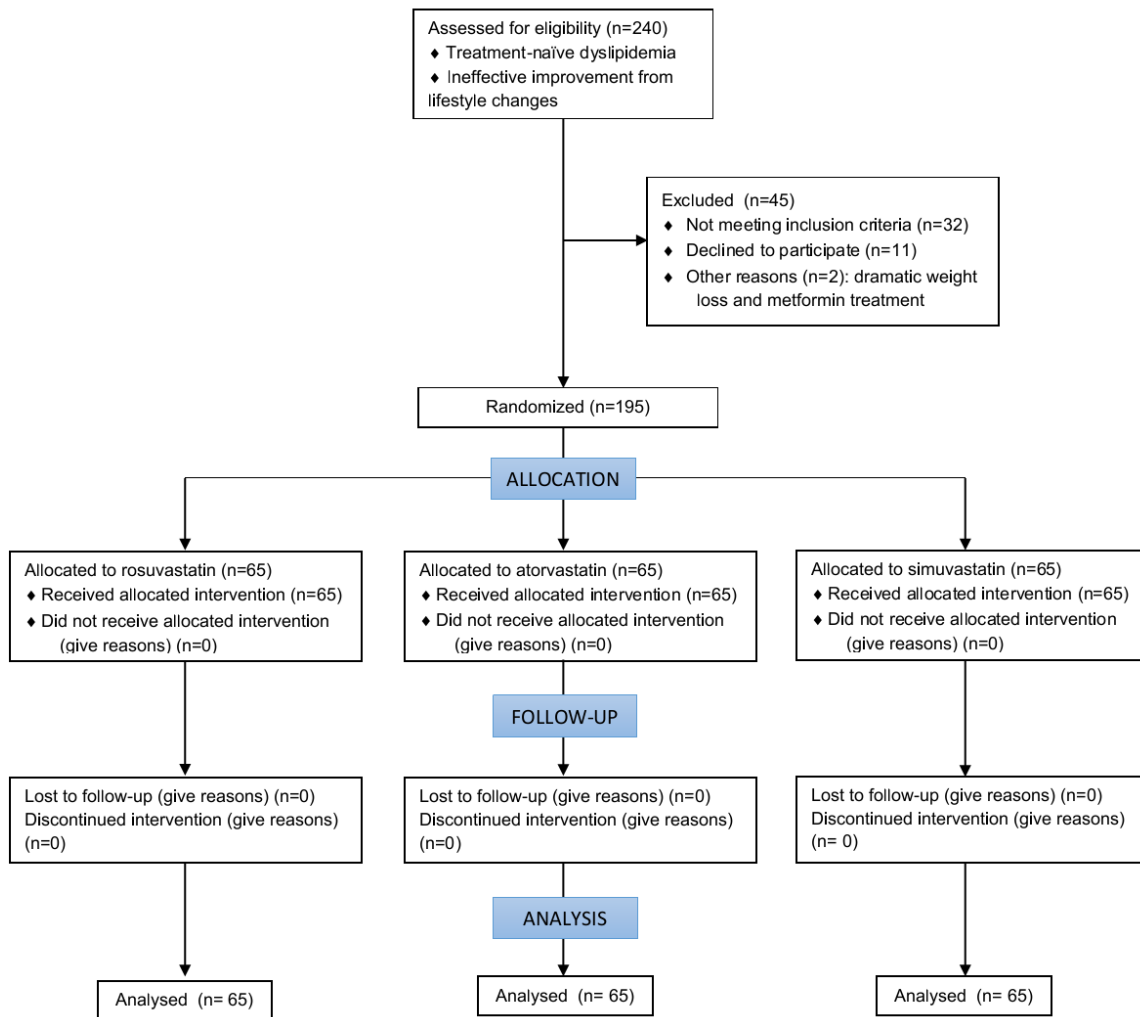

**Supplementary Figure S1.** The flow diagram for this clinical trial.

Supplement: Supplementary file 3 [file Image_1.PDF]
