# Supplementary material for: Influence of APOA5 Locus on the Treatment Efficacy of Three Statins: Evidence From a Randomized Pilot Study in Chinese Subjects
Source: Front Pharmacol. 2018 Apr 11;9:352. doi: 10.3389/fphar.2018.00352 (PMC5904201; doi:10.3389/fphar.2018.00352)
Supplement: Supplementary file 4 [file Image_2.PDF]

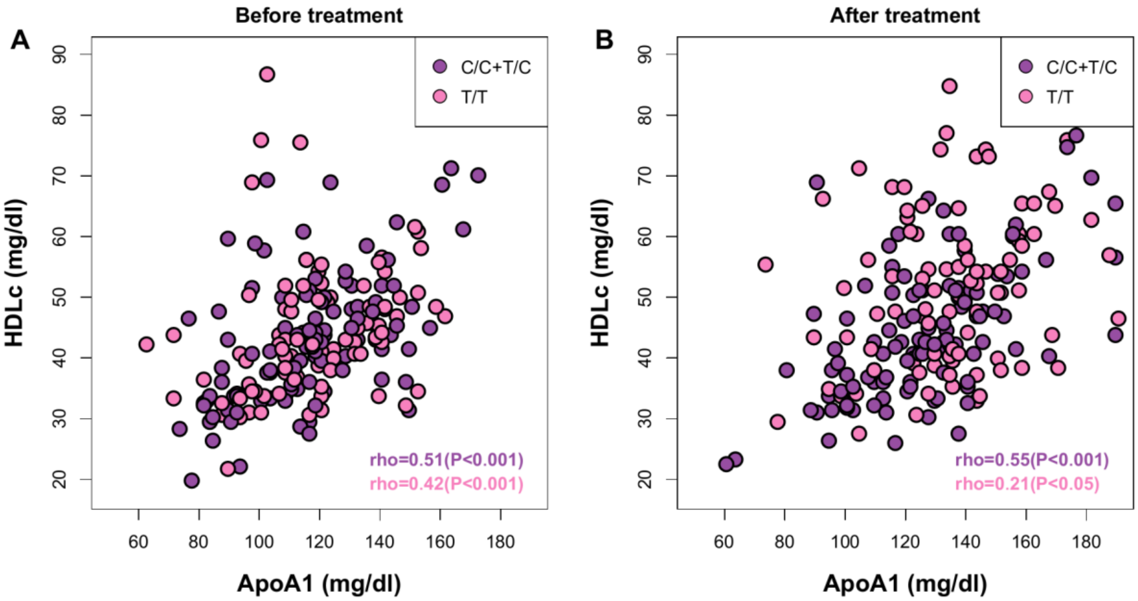

Supplementary Figure S2. Both *APOA5* and statin alter the ApoA1-HDLc correlations. Correlations between ApoA1 and HDLc before (A) and after (B) statin treatment in subjects with *APOA5* rs662799 C or T/T allele, respectively.
